# Efficacy of repetitive transcranial magnetic stimulation with quetiapine in treating bipolar II depression: a randomized, double-blinded, control study

**DOI:** 10.1038/srep30537

**Published:** 2016-07-27

**Authors:** Shao-hua Hu, Jian-bo Lai, Dong-rong Xu, Hong-li Qi, Bradley S. Peterson, Ai-min Bao, Chan-chan Hu, Man-li Huang, Jing-kai Chen, Ning Wei, Jian-bo Hu, Shu-lan Li, Wei-hua Zhou, Wei-juan Xu, Yi Xu

**Affiliations:** 1 Department of Psychiatry, First Affiliated Hospital, Zhejiang University School of Medicine, Hangzhou 310003, China; 2 The Key Laboratory of Mental Disorder’s Management of Zhejiang Province, Hangzhou 310003, China; 3 Zhejiang University School of Medicine, Hangzhou 310058, China; 4 Epidemiology Division & MRI Unit, Department of Psychiatry, Columbia University & New York State Psychiatric Institute, New York 10032, USA; 5 Institute of the Developing Mind, Children’s Hospital Los Angeles, University of Southern California, Los Angeles, California 90027, USA; 6 Department of Neurobiology; Key Laboratory of Medical Neurobiology of Ministry of Health of China; Zhejiang Province Key Laboratory of Neurobiology, Zhejiang University School of Medicine, Hangzhou 310058, China

## Abstract

The clinical and cognitive responses to repetitive transcranial magnetic stimulation (rTMS) in bipolar II depressed patients remain unclear. In this study, thirty-eight bipolar II depressed patients were randomly assigned into three groups: (i) left high-frequency (n = 12), (ii) right low-frequency (n = 13), (iii) sham stimulation (n = 13), and underwent four-week rTMS with quetiapine concomitantly. Clinical efficacy was evaluated at baseline and weekly intervals using the 17-item Hamilton Depression Rating Scale (HDRS-17) and Montgomery-Asberg Depression Rating Scale (MADRS). Cognitive functioning was assessed before and after the study with the Wisconsin Card Sorting Test (WCST), Stroop Word-Color Interference Test (Stroop), and Trail Making Test (TMT). Thirty-five patients were included in the final analysis. Overall, the mean scores of both the HDRS-17 and the MADRS significantly decreased over the 4-week trial, which did not differ among the three groups. Exploratory analyses revealed no differences in factor scores of HDRS-17s, or in response or remission rates. Scores of WCST, Stroop, or TMT did not differ across the three groups. These findings indicated active rTMS combined with quetiapine was not superior to quetiapine monotherapy in improving depressive symptoms or cognitive performance in patients with bipolar II depression.

Bipolar disorder (BpD) is a severe, recurrent, and chronic psychiatric disorder with an estimated population prevalence of 3%^1^. BpD greatly impairs quality of life, social relationship, and occupational performance^2^,^3^. It is associated with cognitive dysfunction, including deficits in verbal learning and expression, working memory, attention and psychomotor speed^4^. Treatment of BpD is challenging due to the high risk of comorbid diseases, the high incidence of residual symptoms and the heavy burden of complex polypharmacy^1^,^5^. Quetiapine monotherapy, lurasidone monotherapy or as an adjuvant to mood stabilizers, and the combination of olanzapine and fluoxetine, have been recommended for the treatment of bipolar depression^6^,^7^. Besides, practitioners may favor the use of antidepressants in patients with bipolar II disorder when response to mood stabilizers is inadequate or the risk of suicide exists^8^. Psychostimulants, followed by modafinil/armodafinil and thyroid hormone, will also be considered for bipolar depression^8^. These psychoactive agents, however, do not significantly improve cognitive functioning, and carry the risk of serious drug-induced side effects^6^,^7^,^8^. Therefore, more effective and safer interventions are needed in managing BpD.

Repetitive transcranial magnetic stimulation (rTMS) has been employed in various neuropsychiatric illnesses, especially for major depressive disorder^9^,^10^. High-frequency rTMS (>5 Hz) delivered to the left dorsolateral prefrontal cortex (DLPFC) and low-frequency rTMS (≤1 Hz) delivered to the right DLPFC both have a neuromodulative effect^11^. However, the clinical efficacy of rTMS in treating depressive symptoms and cognitive deficits in BpD is limited and conflicting. A randomized double-blind trial of 20 depressed BpD patients reported superior improvement in symptoms following two-week active stimulation compared with sham control^12^. An open-label study reported remission in 4 of 11 treatment-resistant patients with bipolar depression, and substantial clinical response in 6 more after a 3-week course of right rTMS (1 Hz, 110% motor threshold)^13^. Moreover, a recent meta-analysis has reported more patients receiving active rTMS achieved clinical responses compared to patients receiving sham rTMS (47/106, 44.3%, vs. 19/75, 25.3%)^14^. Another small randomized controlled trial in 23 patients (21 diagnosed with either bipolar I or II disorder, 2 in a mixed state) found no significant difference following two weeks of left rTMS (5 Hz, 110% motor threshold) and sham stimulation^15^. Negative findings of bilateral rTMS over DLPFC were also exhibited in 49 BpD patients with treatment-resistant depression^16^. In regard to cognitive profiles, significant improvement in cognition was reported in major depressive patients after 20 daily sessions of deep TMS^17^, which was possibly associated to reduction in the depression severity^18^. These studies provide preliminary evidence that rTMS can be safely used in bipolar depressed patients, with a low risk of switching into hypomania/mania^12^,^13^,^14^,^15^,^16^. However, the comparative efficacy of left high-frequency and right low-frequency rTMS in treating depressed BpD patients has yet to be reported. The changes in cognitive performance following rTMS treatment in bipolar patients are not sufficiently elucidated. Of note, the rTMS parameters applied for bipolar depression in above studies, especially its brain target, the DLPFC, was mainly referred to stimulation settings in unipolar depression, regardless of the neuropathological distinctions of these two diseases.

The aim of this study was thus to explore the clinical efficacy and cognitive remediation of rTMS in treating patients with a depressive episode of bipolar II disorder, compare the advantages and disadvantages between left high-frequency and right low-frequency rTMS in reference to its common settings of stimulation parameters in unipolar depression. Given its antidepressant role and preventative effects in treatment-emergent mania or hypomania^6^,^7^, we chose quetiapine as the control condition to ensure that the treatment of all participants was not delayed.

## Results

### Sample characteristics

**Figure 1:**
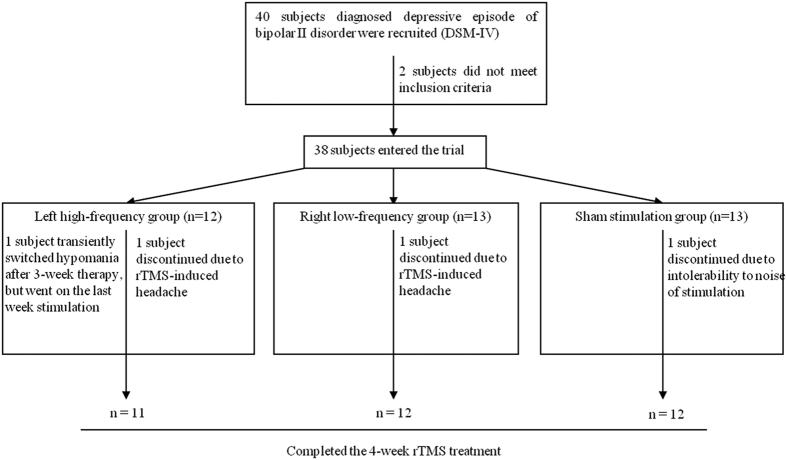
Flowchart of subjects in the rTMS study.

After randomization, 12, 13 and 13 participants entered the left high-frequency, right low-frequency and sham stimulation groups, respectively. The three groups reported withdrawal of one subject each due to intolerable rTMS-induced headache or noise.Thirty-five patients were included in the final analysis. No significant difference was found among the three groups in age, gender, years of education, marital status, onset or duration of illness, or total number of episodes (Table 1). The average dosage of quetiapine was similar across the three groups (*F*_(*2*,*32*)_ = 0.60, *p* = 0.557). A hypomanic switch was detected in one patient in the left high-frequency group after 3 weeks of rTMS therapy (BRMS score of 11). This patient recovered spontaneously in several hours without any medical intervention and successfully completed the 4^th^ week of stimulation. Flowchart of subjects in this study was shown in Fig. 1.

### Clinical outcomes

**Figure 2:**
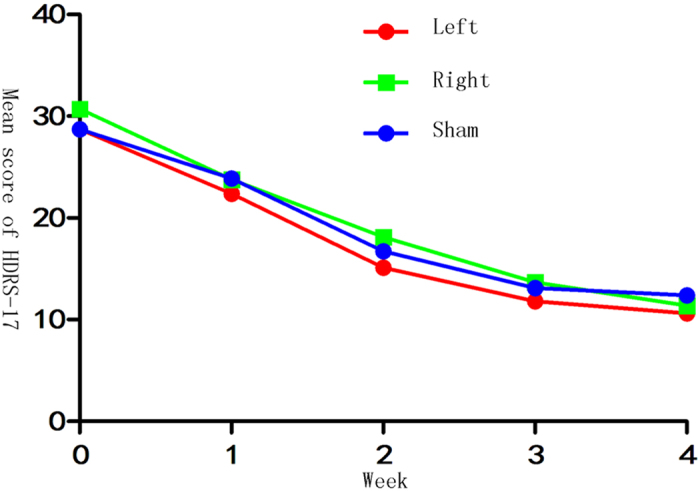
Mean HDRS-17 score of the 3 groups at baseline and at each weekly assessment during the 4-week rTMS trial.

**Figure 3:**
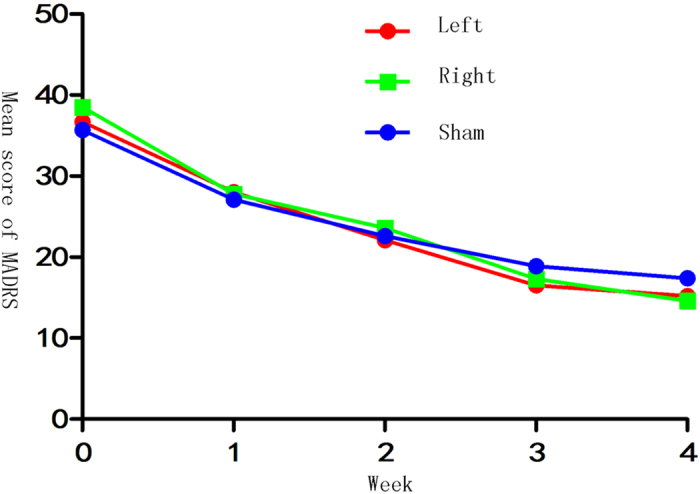
Mean MADRS score of the 3 groups at baseline and at each weekly assessment during the 4-week rTMS trial.

Overall, there was a significant time effect indicating reduction in mean scores of HDRS-17 and MADRS (HDRS-17: *F*_(*2*,*32*)_ = 120.35, *p* < 0.001; MADRS: *F*_(*2*,*32*)_ = 95.66, *p* < 0.001; Greenhouse-Geisser corrected; Figs 2 and 3), but no significant group effect (HDRS-17: *F*_(*2*,*32*)_ = 0.558, *p* = 0.578; MADRS: *F*_(*2*,*32*)_ = 0.039, *p* = 0.962; Figs 2 and 3) or group-by-time interaction (HDRS-17: *F*_(*2*,*32*)_ = 0.299, *p* = 0.892; MADRS: *F*_(*2*,*32*)_ = 0.619, *p* = 0.679; Greenhouse-Geisser corrected; Figs 2 and 3) was observed.

No significant difference in response rates (8/11 vs. 9/12 vs. 8/12, *χ*^2^ = 0.22, *p* = 0.897) or remission rates (3/11 vs. 3/12 vs. 2/12, *χ*^2^ = 0.41, *p* = 0.813) was detected across the three groups.

Meanwhile, all factor scores of the HDRS-17 across the three groups, including anxiety/somatization, cognition, sleep, weight, and retardation, failed to demonstrate any significant difference at baseline and over the 4-week treatment (*p* > 0.05).

### Cognitive outcomes

No statistically significant treatment differences were found in terms of cognitive measures (WCST, Stroop or TMT) across the three groups before and after the rTMS treatment (*p* > 0.05, Table 2).

## Discussion

In the present study, active rTMS with quetiapine compared to quetiapine monotherapy did not appear to be superior in improving the clinical severity of depressive symptoms or in improving cognition in BpD. Neither clinical symptoms (scores on the HDRS-17 and MADRS, and response and remission rates) nor cognitive functioning (WCST, Stroop, and TMT) differed significantly after treatment among the three groups. Similar results were found in exploratory analyses of factor analytic scores of HDRS-17 (anxiety/somatization, cognition, sleep, weight, and retardation).

rTMS is now widely used in clinical practice for the treatment of unipolar depression^9^,^10^,^11^. Compared with unipolar depression, however, the pathophysiology and clinical treatment of BpD is more complex. The role of rTMS in the treatment of depressive episodes in BpD remains unclear. Our study was intended to assess the efficacy of both left high-frequency and right low-frequency rTMS in improving clinical symptoms and executive functioning in patients with depressive episodes of bipolar II disorder during concomitant administration of quetiapine. Prior to our trial, several preliminary studies have explored the role of rTMS in treating BpD, but the studies were in small size, inadequately controlled, and included mood states other than depression^12^,^13^,^14^,^15^. Earlier studies also differ in their parameter settings for rTMS stimulation (10 Hz, 5 Hz or 1 Hz, 110% MT), treatment duration (2, 3 or 4 weeks), and stimulation target (left or right DLPFC). Although several case reports have suggested that rTMS may be useful as an adjunctive acute-phase or maintenance therapy for bipolar depression, further evidence supporting its efficacy is needed^19^,^20^.

The rTMS parameters in our study were selected for consideration of both effectiveness and safety based on previously published guidelines^21^. A subthreshold output intensity of 80% MT was considered safer and more tolerable than supra-stimulation. Transient hypomanic symptoms occurred only in a single case after three weeks of left high-frequency stimulation, and three participants (from three different groups, respectively) withdrew from the study because of rTMS-induced adverse events. The rates of adverse events suggest that rTMS therapy was generally safe and tolerable for patients with a depressive episode in bipolar II disorder. Although no significant differences were detected over time among the three treatment groups, significant reduction in weekly HDRS-17 and MADRS scores was observed, and active rTMS in combination with quetiapine was not superior in efficacy to quetiapine monotherapy. The lack of better efficacy of active stimulation may associate with the location of stimulation. Of note, the region of DLPFC was selected based on extensive neuroimaging evidence suggesting that stimulation of this region was effective in treating unipolar illness^22^,^23^. A recent brain MRI study distinguished unipolar and bipolar depression using a morphometric approach^24^. Subjects with bipolar depression had reduced gray matter volume in the amygdala and hippocampus, and reduced white matter volume in the cerebellum and hippocampus, whereas subjects with unipolar depression contained lower gray matter volume in the anterior cingulate gyrus^24^. These findings indicated that unipolar and bipolar depression may differ neurologically in emotional processing. The neural pathogenesis of unipolar and bipolar depression varies, however, and therefore, the most effective location for stimulation may differ in the depressive phases of these conditions, as well.

Most studies reported no significant differences in cognitive performance between active and sham rTMS^25^. Fewer studies reported the beneficial effects of rTMS in various cognitive domains, such as verbal memory, verbal fluency, motor speed, and global cognitive functioning^25^,^26^,^27^,^28^,^29^. To our knowledge, no previous study has assessed the effects of rTMS on cognitive functioning in bipolar depression. We found no convincing evidence that active rTMS in combination with quetiapine improved executive functioning compared with quetiapine monotherapy. A recent randomized, placebo-controlled study reported no improvement in cognitive or symptom measures in BpD patients after 6 weeks of quetiapine medication. In contrast, the placebo group improved, suggesting that the anticholinergic and antihistaminergic effects of quetiapine perhaps interfered with learning and memory functioning^30^. Therefore, any possible beneficial effects of cognitive restoration in our study could have been obscured by the concomitant administration of quetiapine.

Our study had several limitations: (i) The relatively small sample size limited its statistical power to detect real effects, which also weakened the group difference between left high-frequency and right low-frequency stimulation; (ii) The blinding effectiveness of mood raters was not assessed, which may potentially influence the trial validity and the interpretation of our findings; (iii) Although the average quetiapine doses did not differ significantly across the treatment groups, the dose was not determined before initiation of the study, and the serum concentration of quetiapine was not monitored during the trial. Moreover, the antidepressant role of quetiapine might cover up the true effect of active rTMS on bipolar depression; (iv) We used a relatively low stimulation intensity of 80% of motor threshold and daily stimulation of 1200 pulses for safety reasons, which may have lowered the treatment efficacy. Meanwhile, 4-week daily active rTMS may not be sufficient to achieve apparent clinical outcomes, which possibly require longer stimulation sessions.

In conclusion, as our data demonstrated, active rTMS (either left high-frequency or right low-frequency stimulation) in combination with quetiapine was not superior to quetiapine monotherapy with sham-stimulation in improving either depressive symptoms or executive functioning in patients with a depressive episode of bipolar II disorder. Further investigations with larger sample size are needed to determine the optimal stimulation parameters, including stimulation location and duration of rTMS of bipolar depression.

## Methods

### Participants

This prospective, randomized, and controlled study was approved by the Ethics Committee in the First Affiliated Hospital of Zhejiang University, registered in the Chinese Clinical Trial Registration (ChiCTR-TRC-14004814) on 16/06/2014, an institution belong to the World Health Organization international clinical trial registry platform. The methods were carried out in accordance with the approved guidelines.

Between July, 2014 and July, 2015, we recruited totally 40 patients in this study. Their diagnosis of depressive episodes in bipolar II disorder was ascertained by a senior clinician (N.W.) with the Structured Clinical Interview for DSM-IV axis I (SCID-I). All participants were drug-naïve or had withdrawn from psychoactive medications for at least 1 week prior to commencement of the study. Patients’ baseline total scores exceeded 20 on the 17-item HDRS and ≥4 on the Clinical Global Impression severity of illness scale (CGI-S). Physical examination and routine laboratory tests were performed to exclude the presence of severe medical illnesses or neurologic disorders. Other exclusion criteria included any comorbid psychiatric illness, any form of metal implants, or any history of suicide attempt, drug abuse, seizures, or medications known to lower seizure threshold. Two participants did not meet the inclusion criteria and the remaining 38 participants were randomly assigned in a 1:1:1 ratio to 1 of 3 groups: left high-frequency rTMS, right low-frequency rTMS, or sham stimulation. Prior to the trial, a sequence number was randomly generated for each participant by a computer and a randomization table was also generated. Randomization block, which was constituted by each 6 participants (two were randomly assigned to each group), was used to minimize the imbalance of group sizes. Written informed consent was provided by all participants before commencement of the study. Demographic and clinical profiles are listed in Table 1.

In addition to the assigned rTMS or sham therapy, all patients received quetiapine, which was previously reported to be effective in treating bipolar depression^7^. The dosage of quetiapine was adjusted individually according to the clinical response and side effects in the first two weeks and was maintained till the end of the trial, with the final dose ranging from 200 to 700 mg/d, (Table 1). No other antidepressants, mood stabilizers, first or second generation antipsychotics were allowed during the study.

### rTMS

All rTMS procedures were conducted in a certificated laboratory located in the Department of Mental Health equipped for emergency care. Magnetic stimulation was conducted on a Magstim rapid stimulator (Magstim, Sheffield, UK). The resting motor threshold (MT) was determined by the minimum intensity of magnetic stimulation on the primary motor cortex to elicit five visible muscle contractions out of ten consecutive stimuli in the contralateral abductor pollicis brevis muscle^31^. The figure-eight coil was then positioned in a para-sagittal plane 5.5 cm anterior to the site of MT determination and magnetic stimulation was consequently delivered to the brain region of DLPFC.

In the left high-frequency rTMS group, every treatment session consisted of 30 trains of 4 s stimulation at 10 Hz and 80% MT followed by 12 s off (totaling 1200 pulses per session). In the right low-frequency rTMS group, treatment consisted of 120 trains of 10 s stimulation at 1 Hz and 80% MT followed by 2 s off (also totaling 1200 pulses per session). In the sham-stimulation group, all rTMS parameters were consistent with the left high-frequency rTMS group, except that the coil was positioned vertical to the scalp, thereby eliminating the influence of magnetic field on the cortex^32^. Treatment was administered 5 days each week for 4 consecutive weeks, for a total of 20 sessions.

### Evaluation of clinical outcomes

Depression severity was assessed using the Hamilton Depression Rating Scale (17-item HDRS) at baseline, with the Montgomery-Asberg Depression Rating Scale (MADRS) at weekly intervals throughout the study. A clinical response was defined as a decline in HDRS-17 score greater than 50%; remission was defined as an HDRS-17 score of ≤7. We also compared groups at each time point on the 5 factor scores derived from the HDRS-17: anxiety/somatization, cognition, sleep, weight, and retardation. We used the Beth-Rafaelsen Mania Scale (BRMS) to monitor for any rTMS-induced mania/hypomania symptoms. Two experienced clinicians (J.B.L. & M.L.H.), who were blinded to grouping and stimulation, rated the HDRS-17 and the MADRS, respectively.

### Assessment of cognitive changes

Cognitive functioning was assessed at the beginning and the endpoint of the study using the Chinese modified versions of the Wisconsin Card Sorting Test (WCST)^33^,^34^, Stroop Word-Color Interference Test (Stroop)^35^,^36^, and the Trail Making Test (TMT)^37^. The WCST test was administered using computerized software, whereas the Stroop and TMT were administered manually, by senior clinicians (C.C.H. & J.K.C.) blinded to treatment randomization.

### Statistical analyses

Statistical analyses were conducted using SPSS version 16.0. One-way analysis of variance (ANOVA) was used to analyze measurement data involving sociodemographic and clinical profiles. An Intent-To-Treat method was used to analyze outcome measures (HDRS-17 and factor analytic scores, MADRS, and neuropsychological tests) with repeated-measures ANOVA. Chi-square test was used to compare gender, marital status, response rate, and remission rate across the three treatment groups. A two-tailed *p* < 0.05 was considered statistically significant.

## Additional Information

**How to cite this article**: Hu, S.-H. *et al*. Efficacy of repetitive transcranial magnetic stimulation with quetiapine in treating bipolar II depression: a randomized, double-blinded, control study. *Sci. Rep.* **6**, 30537; doi: 10.1038/srep30537 (2016).

## Figures and Tables

**Table 1:** Sociodemographic and clinical profiles.

Variables	Left high-frequency, n = 11 (mean/n ± SD)	Right low-frequency, n = 12 (mean/n ± SD)	Sham-stimulation, n = 12 (mean/n ± SD)	*χ*^*2*^/*F*_(*2*,*32*)_	p value*
Age/years	27.4 ± 14.3	28.3 ± 10.3	23.7 ± 9.80	0.53	0.595
Gender(Male/Female)	5/6	7/5	5/7	0.73	0.694
Education/years	11.6 ± 2.58	12.7 ± 3.17	10.8 ± 2.55	1.30	0.286
Marital status(Unmarried/married)	7/4	6/6	8/4	0.78	0.676
Age of onset/years	23.7 ± 15.1	24.5 ± 11.0	20.3 ± 9.99	0.41	0.664
Total number of episodes	1.64 ± 0.67	1.92 ± 0.90	3.17 ± 3.04	2.16	0.132
Duration of all episodes/months	47.0 ± 31.5	44.4 ± 30.5	43.3 ± 26.2	0.05	0.954
Doses of quetiapine/mg	372 ± 142	325 ± 129	375 ± 106	0.60	0.557

^*^Significance at *p* < 0.05 (2-tailed).

**Table 2:** Cognitive performance.

Neuropsychological tests	Variables (mean ± SD)	0 = Pre-rTMS 1 = Post-rTMS	Left high-frequency n = 11	Right low-frequency n = 11^a^	Sham n = 12	Group	Group*time
						*F*_(*2*,*31*)_, *p*	*F*_(*2*,*31*)_, *p*
WCST	Number of total trials	0	47.8 ± 0.60	46.6 ± 1.75	47.7 ± 0.62	1.321, 0.281	0.439, 0.649
		1	46.8 ± 2.40	45.4 ± 3.44	45.7 ± 3.47		
	Number of correct trials	0	29.5 ± 6.99	28.4 ± 9.47	29.7 ± 8.90	0.005, 0.995	0.595, 0.558
		1	31.8 ± 4.96	32.4 ± 9.81	31.1 ± 7.82		
	Number of total errors	0	18.4 ± 7.31	18.3 ± 10.9	18.1 ± 9.23	0.038, 0.963	0.292, 0.749
		1	15.0 ± 6.38	13.1 ± 10.3	14.6 ± 10.0		
	Number of preservative errors	0	12.4 ± 5.22	12.3 ± 8.89	11.2 ± 6.50	0.157, 0.856	1.441, 0.252
		1	10.3 ± 4.96	7.45 ± 6.36	9.58 ± 8.06		
	Number of random errors	0	5.91 ± 2.88	6.00 ± 2.97	6.92 ± 3.58	0.151, 0.860	0.457, 0.637
		1	4.73 ± 2.19	5.45 ± 4.61	5.00 ± 3.05		
	Categories completed	0	3.81 ± 1.17	3.55 ± 2.62	3.67 ± 1.87	0.009, 0.991	0.275, 0.761
		1	4.27 ± 1.01	4.36 ± 2.15	4.41 ± 1.88		
Stroop	Part 1	0	50.1 ± 8.50	50.8 ± 15.4	43.7 ± 7.00	0.914, 0.412	1.508, 0.237
		1	42.8 ± 7.78	44.1 ± 8.95	42.5 ± 6.35		
	Part 2	0	90.2 ± 41.6	85.0 ± 20.9	80.8 ± 18.4	0.154, 0.858	0.384, 0.685
		1	76.8 ± 25.3	78.0 ± 28.4	75.4 ± 15.2		
	Part 3	0	152 ± 57.7	140 ± 46.6	119 ± 24.7	1.698, 0.200	0.698, 0.505
		1	137 ± 51.9	114 ± 44.1	111 ± 18.1		
	Interference (Part 3-2)	0	61.5 ± 30.6	55.5 ± 34.1	39.5 ± 19.6	2.823, 0.075	2.208, 0.127
		1	60.5 ± 33.0	35.7 ± 21.7	35.3 ± 15.9		
TMT	Part A	0	75.6 ± 33.1	62.9 ± 18.1	50.7 ± 16.5	1.932, 0.162	2.722, 0.081
		1	53.3 ± 29.1	52.4 ± 16.7	45.3 ± 11.9		
	Part B	0	112 ± 45.7	112 ± 46.9	96.4 ± 27.3	0.642, 0.533	0.311, 0.735
		1	98.2 ± 33.5	89.4 ± 27.8	85.7 ± 26.4		

^*^Significance at p < 0.05 (2-tailed), with repeated-measures analysis of variance. Abbreviations: WCST = Wisconsin Card Sorting Test; Stroop = Stroop Color and Word Test; and TMT = Trail Making Test. ^a^Results of neuropsychological tests of one participant in the right low-frequency group were lost, and sample size of this group decreased to 11.
